# A Survey on Deep Learning for Neuroimaging-Based Brain Disorder Analysis

**DOI:** 10.3389/fnins.2020.00779

**Published:** 2020-10-08

**Authors:** Li Zhang, Mingliang Wang, Mingxia Liu, Daoqiang Zhang

**Affiliations:** ^1^College of Computer Science and Technology, Nanjing Forestry University, Nanjing, China; ^2^College of Computer Science and Technology, Nanjing University of Aeronautics and Astronautics, Nanjing, China; ^3^Department of Radiology and Biomedical Research Imaging Center, University of North Carolina at Chapel Hill, Chapel Hill, NC, United States

**Keywords:** deep learning, neuroimage, Alzheimer's disease, Parkinson's disease, autism spectrum disorder, schizophrenia

## Abstract

Deep learning has recently been used for the analysis of neuroimages, such as structural magnetic resonance imaging (MRI), functional MRI, and positron emission tomography (PET), and it has achieved significant performance improvements over traditional machine learning in computer-aided diagnosis of brain disorders. This paper reviews the applications of deep learning methods for neuroimaging-based brain disorder analysis. We first provide a comprehensive overview of deep learning techniques and popular network architectures by introducing various types of deep neural networks and recent developments. We then review deep learning methods for computer-aided analysis of four typical brain disorders, including Alzheimer's disease, Parkinson's disease, Autism spectrum disorder, and Schizophrenia, where the first two diseases are neurodegenerative disorders and the last two are neurodevelopmental and psychiatric disorders, respectively. More importantly, we discuss the limitations of existing studies and present possible future directions.

## 1. Introduction

Medical imaging refers to several different technologies that are used to provide visual representations of the interior of the human body in order to aid the radiologists and clinicians to detect, diagnose, or treat diseases early and more efficiently (Brody, 2013). Over the past few decades, medical imaging has quickly become a dominant and effective tool and represents various imaging modalities, including X-ray, mammography, ultrasound, computed tomography, magnetic resonance imaging (MRI), and positron emission tomography(PET) (Heidenreich et al., 2002). Each type of these technologies gives various pieces of anatomical and functional information about the different body organs for diagnosis as well as for research. In clinical practice, the detail interpretation of medical images needs to be performed by human experts, such as the radiologists and clinicians. However, for the enormous number of medical images, the interpretations are time-consuming and easily influenced by the biases and potential fatigue of human experts. Therefore, from the early 1980s, doctors and researchers have begun to use computer-assisted diagnosis (CAD) systems to interpret the medical images and to improve their efficiency.

In the CAD systems, machine learning is able to extract informative features that describe the inherent patterns from data and play a vital role in medical image analysis (Wernick et al., 2010; Wu et al., 2016; Erickson et al., 2017; Li et al., 2019). However, the structures of the medical images are very complex, and the feature selection step is still carried out by the human experts on the basis of their domain-specific knowledge. This results in a challenge for non-experts to utilize machine learning techniques in medical image analysis. Therefore, the handcrafted feature selection is not suitable for medical images. Though the sparse learning and dictionary learning have demonstrated the validity of these techniques for automatically discovering discriminative features from training samples, the shallow architectures of these algorithms limit their representational power (Pandya et al., 2019).

Compared to the traditional machine learning algorithms, deep learning automatically discovers the informative representations without the professional knowledge of domain experts and allows the non-experts to effectively use deep learning techniques. Therefore, deep learning has rapidly becomes a methodology of choice for medical image analysis in recent years (LeCun et al., 2015; Schmidhuber, 2015; Goodfellow et al., 2016; Lian et al., 2018). Due to enhanced computer power with the high-tech central processing units (CPU) and graphical processing units (GPU), the availability of big data, and the creation of novel algorithms to train deep neural networks, deep learning has seen unprecedented success in the most artificial intelligence applications, such as computer vision (Voulodimos et al., 2018), natural language processing (Sarikaya et al., 2014), and speech recognition (Bahdanau et al., 2016). Especially, the improvement and successes of computer vision simultaneously prompted the use of deep learning in the medical image analysis (Lee et al., 2017; Shen et al., 2017).

Currently, deep learning has fueled great strides in medical image analysis. We can divide the medical image analysis tasks into several major categories: classification, detection/localization, registration, and segmentation (Litjens et al., 2017). The classification is one of the first tasks in which deep learning giving a major contribution to medical image analysis. This task aims to classify medical images into two or more classes. The stacked auto-encoder model was used to identify Alzheimer's disease or mild cognitive impairment by combining medical images and biological features (Suk et al., 2015). The detection/localization task consists of the localization and identification of the landmarks or lesion in the full medical image. For example, deep convolutional neural networks were used for the detection of lymph nodes in CT images (Roth et al., 2014). The segmentation task is to partition a medical image into different meaningful segments, such as different tissue classes, organs, pathologies, or other biologically relevant structures (Sun et al., 2019a). The U-net was the most well-known deep learning architecture, which used convolutional networks for biomedical image segmentation (Ronneberger et al., 2015). Registration of medical images is a process that searches for the correct alignment of images. Wu et al. (2013) utilized convolutional layers to extract features from input patches in an unsupervised manner. Then the obtained feature vectors were used to replace the handcrafted features in the HAMMER registration algorithm. In addition, the medical image analysis contains other meaningful tasks, such as content-based image retrieval (Li et al., 2018c) and image generation and enhancement (Oktay et al., 2016) in combination with image data and reports (Schlegl et al., 2015).

There are many papers have comprehensively surveyed the medical image analysis using deep learning techniques (Lee et al., 2017; Litjens et al., 2017; Shen et al., 2017). However, these papers usually reviewed all human tissues, including the brain, chest, eye, breast, cardiac, abdomen, musculoskeletal, and others. Almost no papers focus on one specific tissue or disease (Hu et al., 2018). Brain disorders are among the most severe health problems facing our society, causing untold human suffering and enormous economic costs. Many studies successfully used medical imaging techniques for the early detection, diagnosis, and treatment of the human brain disorders, such as neurodegenerative disorders, neurodevelopmental disorders and psychiatric disorders (Vieira et al., 2017; Durstewitz et al., 2019). We therefore pay more close attention to human brain disorders in this survey. About 100 papers are reviewed, most of them published from 2016 to 2019, on deep learning for brain disorder analysis.

The structure of this review can roughly be divided into two parts, the deep learning architectures and the usage of deep learning in brain disorder analysis and is organized as follows. In section 2, we briefly introduce some popular deep learning models. In section 3, we provide a detailed overview of recent studies using deep learning techniques for four brain disorders, including Alzheimer's disease, Parkinson's disease, Autism spectrum disorder, and Schizophrenia. Finally, we analyze the limitations of the deep learning techniques in medical image analysis and provide some research directions for further study. For the convenience of readers, the abbreviations of terminologies used in the following context are listed in the Supplementary Table 1.

## 2. Deep Learning

In this section, we introduce the fundamental concept of basic deep learning models in the literature, which have been wildly applied to medical image analysis, especially human brain disorder diagnosis. These models include feed-forward neural networks, deep generative models (e.g., stacked auto-encoders, deep belief networks, deep Boltzmann machine, and generative adversarial networks), convolutional neural networks, graph convolutional networks, and recurrent neural networks.

### 2.1. Feed-Forward Neural Networks

**Figure 1:**
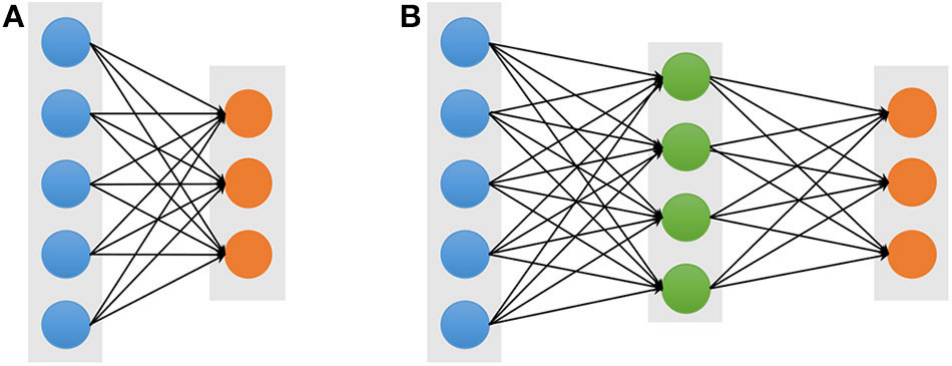
Architectures of the single-layer **(A)** and multi-layer **(B)** neural networks. The blue, green, and orange solid circles represent the input visible, hidden, and output units, respectively.

In machine learning, artificial neural networks (ANN) aim to simulate intelligent behavior by mimicking the way that biological neural networks function. The simplest artificial neural networks is a single-layer architecture, which is composed of an input layer and an output layer (Figure 1A). However, despite the use of non-linear activation functions in output layers, the single-layer neural network usually obtains poor performance for complicated data patterns. In order to circumvent the limitation, the multi-layer perceptron (MLP), also referred to as a feed-forward neural network (FFNN) (Figure 1B), which includes a so-call hidden layer between the input layer and the output layer. Each layer contains multiple units which are fully connected to units of neighboring layers, but there are no connections between units in the same layer. Given an input visible vector ***x***, the composition function of output unit ***y*_*k*_** can be written as follows:

(1) $$\begin{array}{l} y_{k}\left(x;\theta \right)=f^{\left(2\right)}\left(\overset{M}{\underset{j=1}{\sum }}w_{k,j}^{\left(2\right)}f^{\left(1\right)}\left(\overset{N}{\underset{i=1}{\sum }}w_{j,i}^{\left(1\right)}x_{i}+b_{j}^{\left(1\right)}\right)+b_{k}^{\left(2\right)}\right) \end{array}$$

where the superscript represents a layer index, *M* is the number of hidden units, and *b*_*j*_ and *b*_*k*_ represent the bias of input and hidden layer, respectively. *f*^(1)^(·) and *f*^(2)^(·) denote the non-linear activation function, and the parameter set is $\theta =\left\{w_{j}^{\left(1\right)},w_{k}^{\left(2\right)},b_{j}^{\left(1\right)},b_{k}^{\left(2\right)}\right\}$. The back-propagation(BP) is an efficient algorithm to evaluate a gradient in the FFNN (Rumelhart et al., 1986). The BP algorithm is to propagate the error values from the output layer back to the input layer through the network. Once the gradient vector of all the layers is obtained, the parameters **θ** can be updated. Until the loss function is converged or the predefined number of iterations is reached, the update process stops and the network gets the model parameters **θ**.

### 2.2. Stacked Auto-Encoders

**Figure 2:**
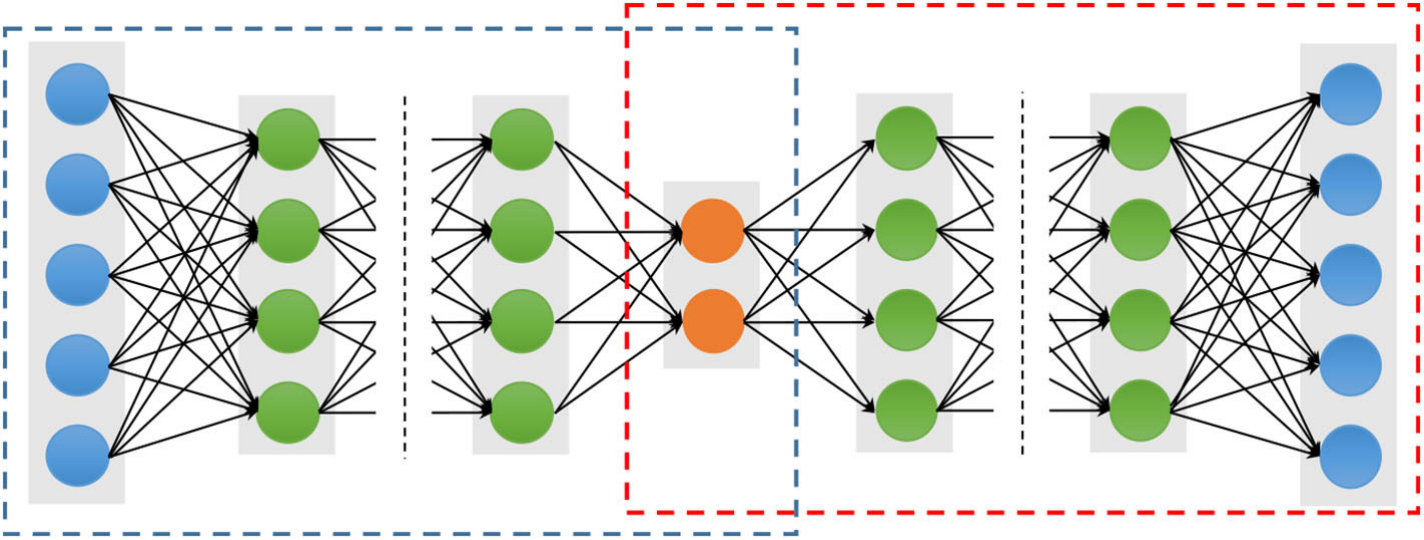
Architectures of a stacked auto-encoder. The blue and red dotted boxes represent the encoding and decoding stage, respectively. The blue solid circles are the input and output units, which have the same number nodes. The orange solid circles represent the latent representation, and the green solid circles represent any hidden layers.

An auto-encoder (AE), also known as an auto-associator, learns the latent representations of input data (called encode) in an unsupervised manner and then uses these representations to reconstruct output data (called decode). Due to the simple and shallow structure, the power representation of a typical AE is relatively limited. However, when multiple AEs are stacked to form a deep network, called stacked auto-encoders (SAE) (Figure 2), the representation power of an SAE can be obviously improved (Bengio et al., 2007). Because of the deep structural characteristic, the SAE is able to learn and discover more complicated patterns inherent in the input data. The lower layers can only learn simpler data patterns, while the higher layers are able to extract more complicated data patterns. In a word, the different layers of an SAE represent different levels of data information (Shen et al., 2017). In addition, various AE variations, denoising auto-encoders (DAE) (Vincent et al., 2008), sparse auto-encoders (sparse AE) (Poultney et al., 2007), and variational auto-encoders (VAE) (Kingma and Welling, 2013), have been proposed and also can be stacked as SAE, such as the stacked sparse AE (SSAE) (Shin et al., 2013). These extensions of auto-encoders not only can learn more useful latent representations but also improve the robustness.

To avoid the drawback of the BP algorithm, which can cause the gradient falling into a poor local optimum (Larochelle et al., 2009), the greedy layer-wise approach is considered to training parameters of an SAE (Hinton and Salakhutdinov, 2006). The important character of the greedy layer-wise is to pre-train each layer in turn. In other words, the output of the *l*-th hidden layers is used as input data for the (*l* + 1)-th hidden layer. The process performs as pre-training, which is conducted in an unsupervised manner with a standard BP algorithm. The important advantage of the pre-training is able to increase the size of the training dataset using unlabeled samples.

### 2.3. Deep Belief Networks

**Figure 3:**
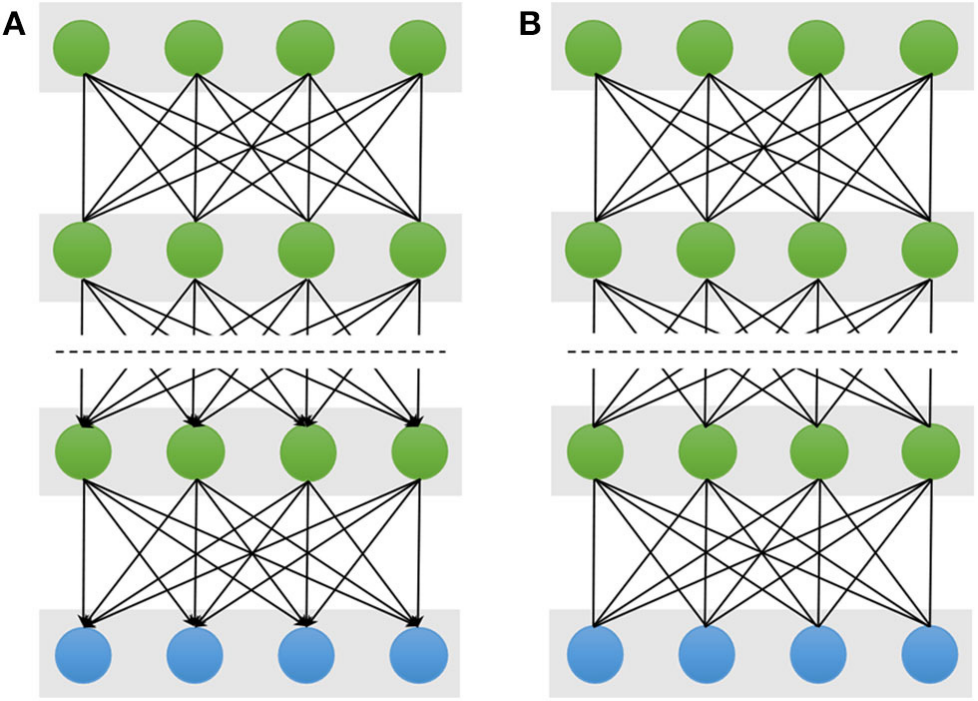
Schematic illustration of Deep Belief Networks **(A)** and Deep Boltzmann Machine **(B)**. The double-headed arrow represents the undirected connection between the two neighboring layers, and the single-headed arrow is the directed connection. The top two layers of the DBN form an undirected generative model and the remaining layers form directed generative model. But all layers of the DBM are undirected generative model.

A Deep Belief Network (DBN) stacks multiple restricted Bolztman machines (RBMs) for deep architecture construction (Hinton et al., 2006). A DBN has one visible layer and multiple hidden layers as shown in Figure 3A. The lower layers form directed generative models. However, the top two layers form the distribution of RBM, which is an undirected generative model. Therefore, given the visible units ***v*** and *L* hidden layers ***h***^(1)^, ***h***^(2)^, …, ***h***^(*L*)^, the joint distribution of DBN is defined:

(2) $$\begin{array}{l} P\left(v,h^{\left(1\right)},\ldots ,h^{\left(L\right)}\right)=P\left(v|h^{\left(1\right)}\right)\left(\overset{L-2}{\underset{l=1}{\prod }}P\left(h^{\left(l\right)}|h^{\left(l+1\right)}\right)\right)P\left(h^{\left(L-1\right)},h^{\left(L\right)}\right) \end{array}$$

where *P*(***h*^(*l*)^|*h*^(*l*+1)^**) represents the conditional distribution for the units of the hidden layer *l* given the units of the hidden layer *l* + 1, and *P*(***h***^(***L*−1**)^, ***h***^(***L***)^) corresponds the joint distribution of the top hidden layers *L* − 1 and *L*.

As for training a DBN, there are two steps, including pre-training and fine-tuning. In the pre-training step, the sDBN is trained by stacking RBMs layer by layer to find the parameter space. Each layer is trained as an RBM. Specifically, the *l*-th hidden layer is trained as an RBM using the observation data from output representation of the (*l* − 1)-th hidden layer, and this repeats, training each layer until the we reach the top layer. After the pre-training is completed, the fine-tuning is performed to further optimize the network to search the optimum parameters. The wake-sleep algorithm and the standard BP algorithm are good at fine-tuning for generative and discriminative models, respectively (Hinton et al., 1995). For a practical application problem, the obtained parameters from the pre-training step are used to initiate a DNN, and then the deep model can be fine-tuned by a supervised learning algorithm like BP.

### 2.4. Deep Boltzmann Machine

A Deep Boltzmann Machine (DBM) is also constructed by stacking multiple RBMs as shown in Figure 3B (Salakhutdinov and Larochelle, 2010; Salakhutdinov, 2015). However, unlike the DBN, all the layers of the DBM form an entirely undirected model, and each variable within the hidden layers are mutually independent. Thus, the hidden layer *l* is conditioned on its two neighboring layer *l* − 1 and *l* + 1, and its probability distribution is *P*(***h***^(*l*)^|***h***^(*l*−1)^, ***h***^(*l*+1)^). Given the values of the neighboring layers, the conditional probabilities over the visible and the *L* set of hidden units are given by logistic sigmoid functions:

(3) $$\begin{array}{l} P\left(v_{i}|h^{1}\right)=\sigma \left(\underset{j}{\sum }W_{ij}^{\left(1\right)}h_{j}^{\left(1\right)}\right) \end{array}$$

(4) $$\begin{array}{l} P\left(h_{k}^{\left(l\right)}|h^{\left(l-1\right)},h^{\left(l+1\right)}\right)=\sigma \left(\underset{m}{\sum }W_{mk}^{\left(l\right)}h_{m}^{\left(l-1\right)}+\underset{n}{\sum }W_{kn}^{\left(l+1\right)}h_{n}^{\left(l+1\right)}\right) \end{array}$$

(5) $$\begin{array}{l} P\left(h_{t}^{\left(L\right)}|h^{\left(L-1\right)}\right)=\sigma \left(\underset{s}{\sum }W_{st}^{\left(L\right)}h_{s}^{\left(L-1\right)}\right) \end{array}$$

Note that in the computation of the conditional probability of the hidden unit ***h***^(*l*)^, the probability incorporate both the lower hidden layer ***h***^(*l*−1)^ and the upper hidden layer ***h***^(*l*+1)^. Due to incorporate the more information from the lower and upper layers, the representational power of a DBM is more robust in the face of the noisy observed data (Karhunen et al., 2015). However, the character makes the conditional probability of DBM *P*(***h***^(*l*)^|***h***^(*l*−1)^, ***h***^(*l*+1)^) more complex than those of the DBN, *P*(***h***^(*l*)^|***h***^(*l*+1)^).

### 2.5. Generative Adversarial Networks

**Figure 4:**
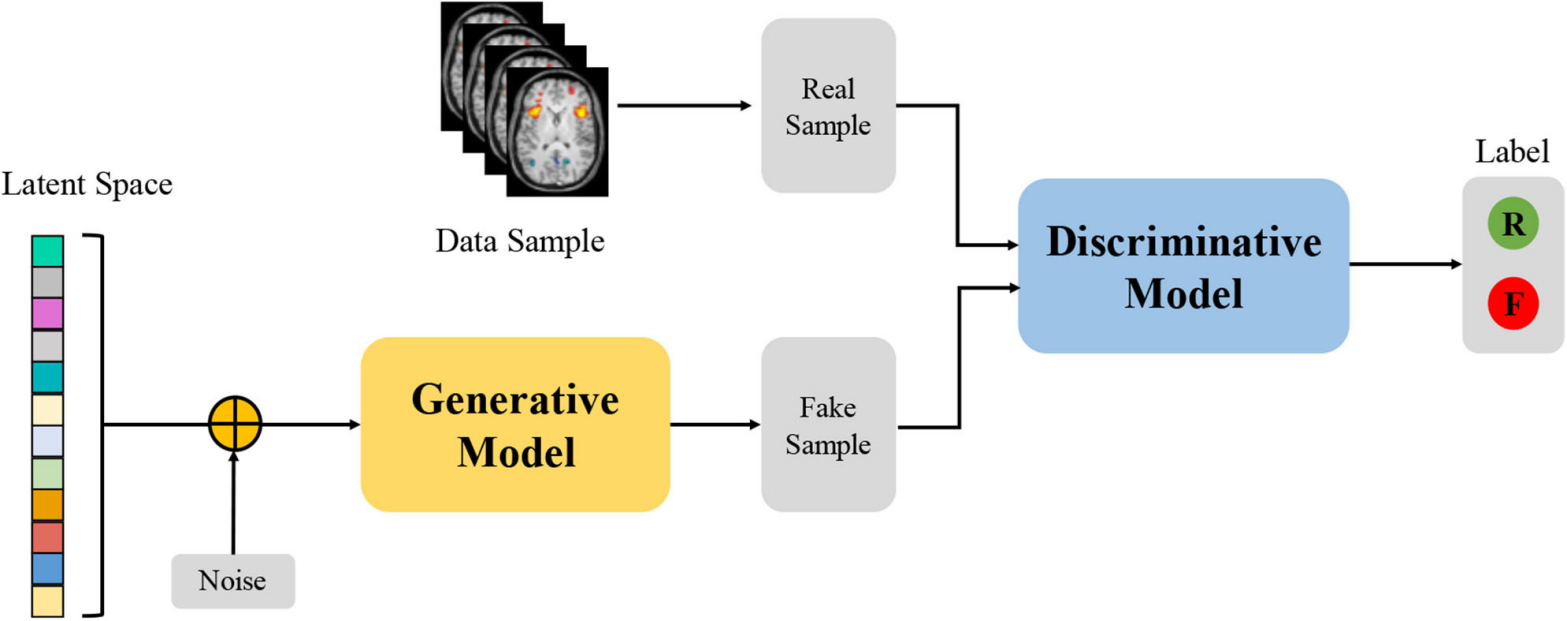
Architecture of Generative Adversarial Networks. “R” and “F” represents the real and fake label, respectively.

Due to their ability to learn deep representations without extensively annotated training data, Generative Adversarial Networks (GANs) have gained a lot of attention in computer vision and natural language processing (Goodfellow et al., 2014). GANs consist of two competing neural networks, a generator *G* and a discriminator *D*, as shown in Figure 4. The generator *G* parameterized by **θ** takes as input a random noise vector ***z*** from a prior distribution *p*_*z*_(***z***; **θ**) and outputs a sample *G*(***z***), which can be regarded as a sample drawn from the generator data distribution *p*_*g*_. The discriminator *D* that takes an input *G*(***z***) or ***x***, and outputs the probability *D*(***x***) or *D*(***G***(***z***)) to evaluate that the sample is from the generator *G* or the real data distribution. GANs simultaneously train the generator and discriminator where the generator *G* tries to generate realistic data to fool the discriminator, while the discriminator *D* tries to distinguish between the real and fake samples. Inspired by the game theory, the training process is to form a two-player minimax game with the value function *V*(*G, D*) as follow:

(6) $$\begin{array}{l} \underset{G}{\min}\underset{D}{\max}V\left(G,D\right)={𝔼}_{x\sim p_{data}\left(x\right)}\left[\text{log}D\left(x\right)\right]\\ \;+\;{𝔼}_{z\sim p_{z}\left(z\right)}\left[\text{log}\left(1-D\left(G\left(z\right)\right)\right)\right] \end{array}$$

where *p*_*data*_(***x***) denotes the real data distribution. After training alternately, if *G* and *D* have enough capacity, they will reach a point at which both cannot improve because *p*_*g*_ = *p*_*data*_. In other words, the discriminator is unable to distinguish the difference between a real and a generated sample, i.e., *D*(***x***) = 0.5. Although vanilla GAN has attracted considerable attention in various applications, there still remain several challenges related to training and evaluating GANs, such as model collapse and saddle points (Creswell et al., 2018). Therefore, many variants of GAN, such as Wasserstein GAN (WGAN) (Arjovsky et al., 2017) and Deep Convolutional GAN (DCGAN) (Radford et al., 2015) have been proposed to overcome these challenges.

### 2.6. Convolutional Neural Networks

**Figure 5:**
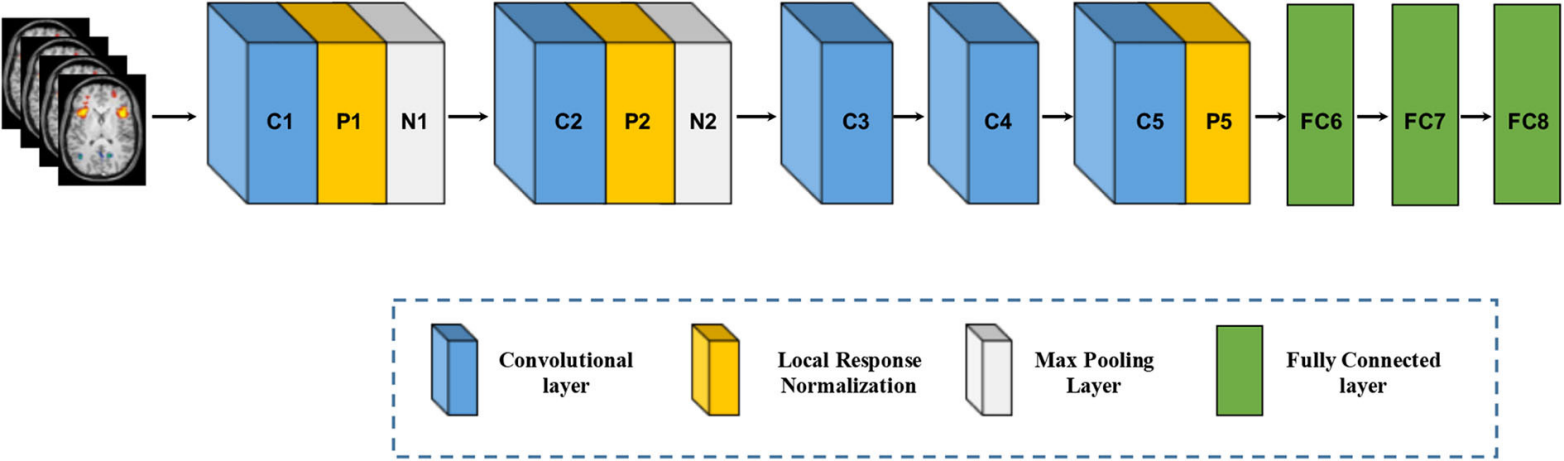
Architecture of convolutional neural networks. Note that an implicit rectified linear unit (ReLU) non-linearity is applied after every layer. The natural images as input data in Krizhevsky et al. (2012) are replaced by brain MR images.

Compared to the SAE, DBN, and DBM, utilizing the inputs in vector form which inevitably destroys the structural information in images, the convolutional neural network (CNN) is designed to better retain and utilize the structural information among neighboring pixels or voxels and to required minimal preprocessing by directly taking two-dimensional (2D) or three-dimensional (3D) images as inputs (LeCun et al., 1998). Structurally, a CNN is a sequence of layers, and each layer of the CNN transforms one volume of activations to another through a differentiable function. Figure 5 shows a typical CNN architecture (AlextNet model) for a computer vision task, which consists of three type neural layers: convolutional layers, pooling layers and fully connected layers (Krizhevsky et al., 2012). The convolutional layers are interspersed with pooling layers, eventually leading to the fully connected layers. The convolutional layer takes the pixels or voxels of a small patch of the input images, called the local receptive field and then utilizes various learnable kernels to convolve the receptive field to generate multiple feature maps. A pooling layer performs the non-linear downsampling to reduce the spatial dimensions of the input volume for the next convolutional layer. The fully connected layer input the 3D or 2D feature map to a 1D feature vector. The local response normalization is a non-trainable layer and performs a kind of “lateral inhibition” by normalizing over local input regions.

The major issue in training deep models is the over-fitting, which arises from the gap between the limited number of training samples and a large number of learnable parameters. Therefore, various techniques are designed to make the models train and generalize better, such as dropout and batch normalization to just name a few. A dropout layer randomly drops a fraction of the units or connections during each training iteration (Srivastava et al., 2014). It has also been demonstrated that dropout is able to successfully avoid over-fitting. In addition, batch normalization is another useful regularization and performs normalization with the running average of the mean–variance statistics of each mini-batch. It is shown that using batch normalization not only drastically speeds up the training time but also improves the generalization performance (Ioffe and Szegedy, 2015).

### 2.7. Graph Convolutional Networks

**Figure 6:**
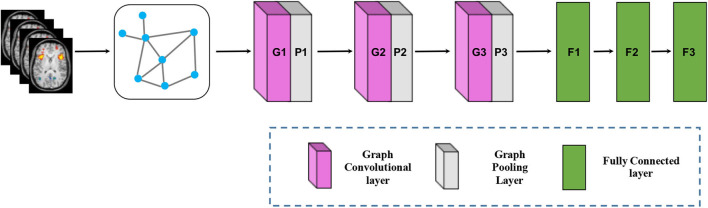
Architecture of graph convolutional networks. To keep the figure simple, the softmax output layer is not shown.

While the CNN has achieved huge success in extracting latent representations from Euclidean data (e.g., images, text, and video), there are a rapidly increasing number of various applications where data are generated from the non-Euclidean domain and needs to be efficiently analyzed. Researchers straightforwardly borrow ideas from CNN to design the architecture of graph convolutional networks (GCN) to handle complexity graph data (Kipf and Welling, 2016). Figure 6 shows the process of a simple GCN with graph pooling layers for a graph classification task. The first step is to transform the traditional data to graph data, and the graph structure and node content information are therefore regarded as input. The graph convolutional layer plays a central role in extracting node hidden representations from aggregating the feature information from its neighbors. The graph pooling layers can be interleaved with the GCN layers and coarsened graphs into sub-graphs in order to obtained higher graph-level representations for each node on coarsened sub-graphs. After multiple fully connected layers, the softmax output layer is used to predict the class labels.

Depending on the types of graph convolutions, the GCN can be categorized into spectral-based and spatial-based methods. Spectral-based methods formulated graph convolution by introducing filters from the perspective of graph single processing. Spatial-based methods defined graph convolution directly on the graph, which operates on spatial close neighbors to aggregate feature information. Due to drawbacks to spectral-based methods from three aspects, efficiency, generality, and flexibility, spatial-based methods have attracted more attention recently (Wu et al., 2019).

### 2.8. Recurrent Neural Networks

**Figure 7:**
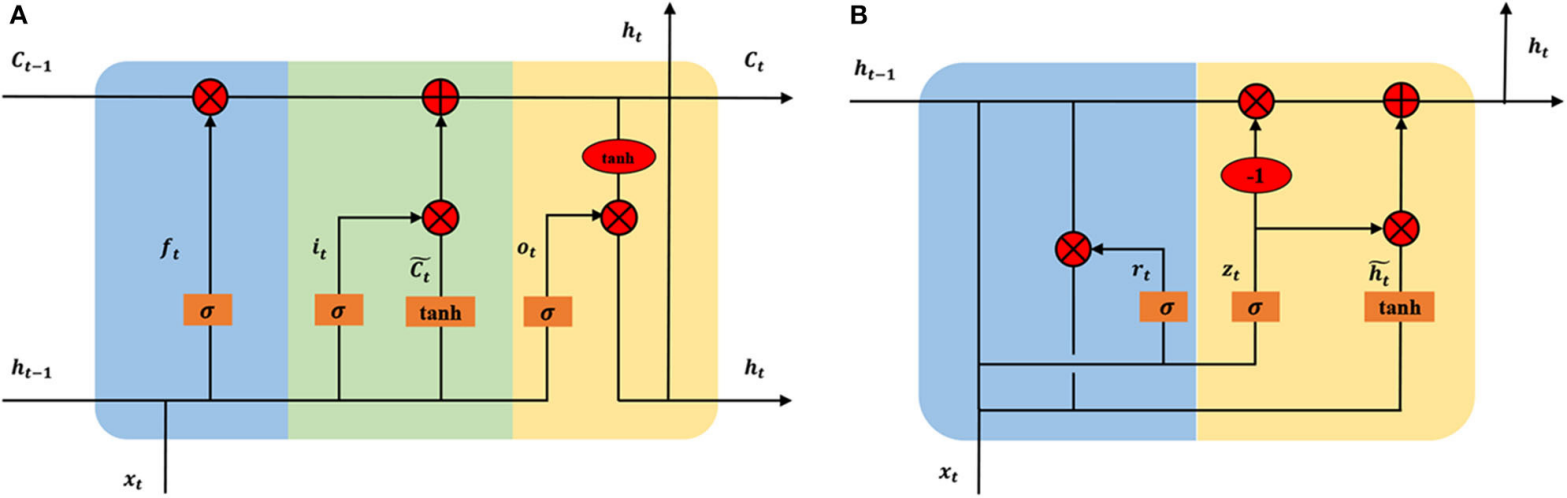
Architectures of long short-term memory **(A)** and gated recurrent unit **(B)** In the subfigure **(A)**, the blue, green, and yellow represent the forget gate *f*_*t*_, input, gate *i*_*t*_, and output gate *o*_*t*_, respectively. In the subfigure **(B)**, the blue and yellow represent the reset gate *r*_*t*_ and update gate *z*_*t*_, respectively. *x*_*t*_ is input vector and *h*_*t*_ is the hidden state. To keep the figure simple, biases are not shown.

A recurrent neural network (RNN) is an extension of an FFNN, which is able to learn features and long-term dependencies from sequential and time-series data. The most popular RNN architecture is the long-short-term memory (LSTM) (Hochreiter and Schmidhuber, 1997), which is composed of a memory cell *C*_*t*_, a forget gate *f*_*t*_, an input gate *i*_*t*_, and an output gate *o*_*t*_ (Figure 7A). The memory cell transfers relevant information all the way to the sequence chain, and these gates control the activation signals from various sources to decide which information is added to and removed from the memory cell. Unlike a basic RNN, the LSTM is able to decide whether to preserve the existing memory by the above-introduced gates. Theoretically, if the LSTM learns an important feature from the input sequential data, it can keep this feature over a long time, thus captures potential long-time dependencies. One popular LSTM variant is the Gated Recurrent Unit (GRU) (Figure 7B), which merges the forget and input gates into a single “update gate,” and combines the memory cell state and hidden state into one state. The update gate decides how much information to add and throw away, and the reset gate decides how much previous information to forget. This makes the GRU is simpler than the standard LSTM (Cho et al., 2014).

### 2.9. Open Source Deep Learning Library

With the great successes of deep learning techniques in various applications, some famous research groups and companies have released their source codes and tools in deep learning. Due to these open source toolkits, people are able to easily build deep models for their applications even if they are not acquainted with deep learning techniques. Supplementary Table 2 lists the most popular toolkits for deep learning and shows their main features.

## 3. Applications in Brain Disorder Analysis With Medical Images

The human brain is susceptible to many different disorders that strike at every stage of life. Developmental disorders usually first appear in early childhood, such as autism spectrum disorder and dyslexia. Although psychiatric disorders are typically diagnosed in teens or early adulthood, their origins may exist much earlier in life, such as depression and schizophrenia. Then, as people age, people become increasingly susceptible to Alzheimer's disease, Parkinson's disease, and other dementia diseases. In this section, we select four typical brain disorders, including Alzheimer's disease, Parkinson's disease, Autism spectrum disorder and Schizophrenia. Alzheimer's disease and Parkinson's disease are both neurodegenerative disorders. Autism spectrum disorder and Schizophrenia are neurodevelopmental and psychiatric disorders, respectively.

### 3.1. Deep Learning for Alzheimer's Disease Analysis

Alzheimer's disease (AD) is a neurological, irreversible, progressive brain disorder and is the most common cause of dementia. Until now, the causes of AD are not yet fully understood, but accurate diagnosis of AD plays a significant role in patient care, especially at the early stage. For the study of AD diagnosis, the best-known public neuroimaging dataset is from the Alzheimer's Disease Neuroimaging Initiative (ADNI), which is a multi-site study that aims to improve clinical trials for the prevention and treatment of AD. The ADNI study has been running since 2004 and is now in its third phase (Mueller et al., 2005). Researchers collect, validate, and utilize data, including MRI and PET images, genetics, cognitive tests, cerebrospinal fluid (CSF), and blood biomarkers as predictors of the disease. Up to now, the ADNI dataset consists of ADNI-1, ADNI-GO, ADNI-2, and ADNI-3 and contains more than 1,000 patients. According to the Mini-Mental State Examination (MMSE) scores, these patients were in three stages of disease: normal control (NC), mild cognitive impairment (MCI), and AD. The MCI subject can be divided into two subcategories: converted MCI (cMCI) and stable MCI (sMCI), based on whether a subject converted to AD within a period of time (e.g., 24 months). The ADNI-GO and ADNI-2 provided two different MCI groups: early mild cognitive impairment (EMCI) and late mild cognitive impairment (LMCI), determined by a Wechsler Memory Scale (WMS) neuropsychological test.

Recently, plenty of papers have been published on the deep learning techniques for AD diagnosis. According to different architectures, these methods can be roughly divided into two subcategories: DGM-based and CNN-based methods. The DGM-based methods contained the DBN, DNM, SAE, and AE variants. Li et al. (2015) stacked multiple RBMs to construct a robust deep learning framework, which incorporated the stability selection and the multi-task learning strategy. Suk et al. (2014) proposed a series of methods based on deep learning models, such as the DBM and SAE (Suk et al., 2015, 2016). For example, the literature (Suk et al., 2015) applied the SAE to learn the latent representations from sMRI, PET, and CSF, respectively. Then, a multi-kernel SVM classifier was used to fuse the selected multi-modal features. Liu et al. (2015) also used SAE to extract features from multi-modal data, and a zero-masking strategy was then applied to fuse these learned features. Shi et al. (2017a) adopted multi-modality stacked denoising sparse AE (SDAE) to fuse cross-sectional and longitudinal features estimated from MR brain images. Lu et al. (2018) developed a multiscale deep learning network, which took the multiscale patch-wise metabolism features as input. This study was perhaps also the first study to utilize such a large number of FDG-PET images data. Martinez-Murcia et al. (2019) used a deep convolution AE (DCAE) architecture to extract features, which showed large correlations with clinical variables, such as age, tau protein deposits, and especially neuropsychological examinations. Due to small labeled samples in neuroimaging dataset, Shi et al. (2017b) proposed a multimode-stacked deep polynomial network (DPN) to effectively fuse and learn feature representation from a small multimodel neuroimaging data.

CNN-based methods learned all levels of features from raw pixels and avoided the manual ROIs annotation procedure and can be further subdivided into two subcategories: 2D-CNN and 3D-CNN. Gupta et al. (2013) pre-trained a 2D-CNN based on sMRI data through a sparse AE on random patches of natural images. The key technique was the use of cross-domain features to present MRI data. Liu and Shen (2014) used a similar strategy and pre-trained a pre-trained deep CNN on ImageNet. Sarraf et al. (2016) first used the fMRI data in deep learning applications. The 4D rs-fMRI and 3D MRI data were decomposed into 2D format images in the preprocessing step, and then the CNN-based architecture received these images in its input layer. Billones et al. designed a DemNet model based on the 16-layer VGGNet. The DemNet only selected the coronal image slices with indices 111–130 in 2D format images under the assumption that these slices covered the areas, which had the important features for the classification task (Billones et al., 2016). Liu et al. (2018b) proposed a novel classification framework that learned features from a sequence of 2D slices by decomposing 3D PET images. Then hierarchical 2D-CNN was built to capture the intra-slice features, while GRU was adopted to extract the inter-slice features.

The 3D brain images need to be decomposed into 2D slices in the preprocessing step, and this results in 2D-CNN methods discarding the spatial information. Many 3D-CNN methods were therefore proposed, and these can directly input 3D brain images. Payan and Montana (2015) pre-trained a 3D-CNN through a sparse AE on small 3D patches from sMRI scans. Hosseini-Asl et al. (2016) proposed a deep 3D-CNN, which was built upon a 3D CAE (Convolutional AE) to capture anatomical shape variations in sMRI scans. Liu et al. used multiple deep 3D-CNN on different local image patches to learn the discriminative features of MRI and PET images. Then, a set of upper high-level CNN was cascaded to ensemble the learned local features and discovered the latent multi-modal features for AD classification (Liu et al., 2018a). Karasawa et al. (2018) proposed deeper 3D-CNN architecture with 39 layers based on a residual learning framework (ResNet) to improve performance. Liu et al. (2018d) designed a landmark-based deep feature learning framework to learn the patch-level features, which were an intermediate scale between voxel-level and ROI-level. The authors firstly used a data-driven manner to identify discriminative anatomical landmarks from MR images, and they then proposed a 3D-CNN to learn patch-based features. This strategy can avoid the high-dimensional problem of voxel-level and manual definition of ROI-level. Subsequently, Liu et al. (2018c) developed a deep multi-instance CNN framework, where multiple image patches were used as a bag of instances to represent each specific subject, and then the label of each bag was given by the whole-image-level class label. To overcome the missing modality in multi-modal image data, Li et al. (2014) proposed a simple 3D-CNN to predict the missing PET images from the sMRI data. Results showed that the predicted PET data achieved similar classification accuracy to the true PET data. Additionally, the synthetic PET data and the real sMRI data obviously outperformed the single sMRI data. Pan et al. (2018) used Cycle-GAN to learn bi-directional mapping sMRI and PET to synthesize missing PET scans based on its corresponding sMRI scans. Then, landmark-based 3D-CNN was adapted for AD classification on the mixed image data. Tables 1, 2 summarized the statistic information of each paper reviewed above for AD diagnosis.

**Table 1:** Overview of papers using deep learning techniques for AD diagnosis.

**References**	**Year**	**Database**	**Subjects**				**Modality**	**Model**
			**AD**	**cMCI**	**sMCI**	**NC**		
Suk et al. (2014)	2014	ADNI	93	76	128	101	sMRI + PET	DBM
Li et al. (2015)	2015	ADNI	51	43	56	52	sMRI + PET + CSF	DBN
Liu et al. (2015)	2015	ADNI	85	67	102	77	sMRI + PET	SAE
Suk et al. (2015)	2015	ADNI	51	43	56	52	sMRI + PET + CSF	SAE
Suk et al. (2016)	2016	ADNI	51	43	56	52	sMRI + PET + CSF	SAE
		–	198	167	236	229		
Shi et al. (2017a)	2017	ADNI	95	121		123	sMRI + Age	SDAE
Shi et al. (2017b)	2017	ADNI	51	43	56	52	sMRI + PET	DPN
Lu et al. (2018)	2018	ADNI	226	112	409	304	PET	SAE
Martinez-Murcia et al. (2019)	2019	ADNI	99	212		168	rs-fMRI	DCAE
Gupta et al. (2013)	2013	ADNI	200	411		232	sMRI	2D-CNN
Liu and Shen (2014)	2014	ADNI	200	411		232	sMRI	2D-CNN
Billones et al. (2016)	2016	ADNI	300	300		300	rs-fMRI	2D-CNN
Sarraf et al. (2016)	2016	ADNI	211	–	–	91	sMRI	2D-CNN
			52	–	–	92	rs-fMRI	
Liu et al. (2018b)	2017	ADNI	93	146		100	PET	2D-CNN + RNN
Payan and Montana (2015)	2015	ADNI	755	755		755	sMRI	3D-CNN
Hosseini-Asl et al. (2016)	2016	ADNI	70	70		70	sMRI	3D-CNN
Karasawa et al. (2018)	2018	ADNI	348	450	358	574	sMRI	3D-CNN
Liu et al. (2018a)	2018	ADNI	93	76	128	100	sMRI + PET	3D-CNN
Li et al. (2014)	2014	ADNI	193	167	236	229	sMRI + PET	3D-CNN
Liu et al. (2018c)	2018	ADNI	358	205	465	429	sMRI	3D-CNN
Liu et al. (2018d)	2018	ADNI	358	–	–	429	sMRI	3D-CNN
Pan et al. (2018)	2018	ADNI	358	205	465	429	sMRI + PET	3D-CNN + GAN

**Table 2:** The classification performance of papers for AD diagnosis.

**References**	**Accuracy (%)**					
	**AD/NC**	**AD/MCI**	**MCI/NC**	**cMCI/sMCI**	**3-ways^**a**^**	**4-ways^**b**^**
Suk et al. (2014).	95.35 ± 5.23	–	85.67 ± 5.22	75.92 ± 15.37	–	–
Li et al. (2015)	91.4 ± 1.8	70.1 ± 2.3	77.4 ± 1.7	57.4 ± 3.6		
Liu et al. (2015)	91.4 ± 5.56	–	82.10 ± 4.91	–	–	53.79
Suk et al. (2015)	98.8 ± 0.9	83.7 ± 1.5	90.7 ± 1.2	83.3 ± 2.1	–	–
Suk et al. (2016)	95.09 ± 2.28	–	80.11 ± 2.64	74.15 ± 3.35	62.93	53.72
	90.27	–	70.86	73.93	57.74	47.83
Shi et al. (2017a)	91.95 ± 1.00	–	83.72 ± 1.16	–	–	–
Shi et al. (2017b)	97.13 ± 4.44	–	87.24 ± 4.52	76.88 ± 4.38	–	57.0 ± 3.65
Lu et al. (2018)	93.58 ± 5.2	–	–	81.55 ± 7.42	–	–
Martinez-Murcia et al. (2019)	84.3 ± 6	–	–	71.5 ± 9	–	–
Gupta et al. (2013)	94.74	88.10	86.35	–	85.0	–
Liu and Shen (2014)	97.18 ± 1.5	94.51 ± 1.43	93.21 ± 1.02	–	91.72 ± 1.8	–
Billones et al. (2016)	98.33	93.89	91.67	–	91.85	–
Sarraf et al. (2016)	98.84/99.90	–	–	–	–	–
Liu et al. (2018b)	91.92	–	78.9	–	–	–
Payan and Montana (2015)	95.39	86.84	92.11	–	89.47	–
Hosseini-Asl et al. (2016)	99.3 ± 1.6	100	94.2 ± 2.0	–	94.8 ± 2.6	–
Karasawa et al. (2018)	94.0	–	90.0	–	87.0	–
Liu et al. (2018a)	93.26	–	73.34	–	–	–
Li et al. (2014)	92.87 ± 2.07	–	76.21 ± 2.05	72.44 ± 2.41	–	–
Liu et al. (2018c)	91.09	–	–	76.90	–	–
Liu et al. (2018d)	90.56	–	–	–	–	–
Pan et al. (2018)	92.50	–	–	79.06	–	–

a *3-ways represents the comparison: AD vs. NC vs. MCI*.

b *4-ways represents the comparison: AD vs. NC vs. cMCI vs. sMCI*.

As an early stage of AD, MCI had a conversion rate as high as 10–15% per year in 5 years, but MCI was also the best time for treatment. Therefore, an effective predictive model construction for the early diagnosis of MCI had become a hot topic. Recently, some research based on GCN has been done for MCI prediction. Yu et al. (2019) and Zhao et al. (2019) both used the GCN, which combines neuroimaging information and the demographic relationship for MCI prediction. Song et al. (2019) implemented a multi-class the GCN classifier for classification of subjects on the AD spectrum into four classes. Guo et al. (2019) proposed PETNET model based on the GCN to analyzes PET signals defined on a group-wise inferred graph structure. Tables 3, 4 summarized the four papers for MCI prediction.

**Table 3:** Overview of papers using deep learning techniques for MCI prediction.

**References**	**Year**	**Database**	**Subjects**				**Modality**	**Model**
			**NC**	**EMCI**	**LMCI**	**AD**		
Zhao et al. (2019)	2019	ADNI	67	77	40	–	rs-fMRI	GCN
Yu et al. (2019)	2019	ADNI	44	44	38	–	rs-fMRI	GCN
Song et al. (2019)	2019	ADNI	12	12	12	12	DTI	GCN
Guo et al. (2019)	2019	ADNI	100	96	137	–	PET	GCN

**Table 4:** The classification performance of papers for MCI prediction.

**References**	**Accuracy (%)**					
	**EMCI/NC**	**LMCI/NC**	**EMCI/LMIC**	**MCI/NC**	**3-ways^**a**^**	**4-ways^**b**^**
Zhao et al. (2019)	78.4	84.3	85.6	–	–	–
Yu et al. (2019)	87.5	89.02	79.27	–	–	–
Song et al. (2019)	–	–	–	–	–	89.0 ± 6
Guo et al. (2019)	–	–	–	93.0^c^	77.0	–

a *3-ways represents the comparison: NC vs. EMCI vs. LMCI*.

b *4-ways represents the comparison: NC vs. EMCI vs. LMCI vs. AD*.

c *MCI = ECMI + LMCI*.

### 3.2. Deep Learning for Parkinson's Disease Analysis

Parkinson's disease (PD) is the most common neurodegenerative disorder after Alzheimer's disease, and it is provoked by progressive impairment and deterioration of neurons, caused by a gradually halt in the production of a chemical messenger in the brain. Parkinson's Progression Markers Initiative (PPMI) is an observational clinical study to verify progression markers in Parkinson's disease. The PPMI cohort comprises 400 newly diagnosed PD cases, 200 healthy, and 70 individuals that, while clinically diagnosed as PD cases, fail to show evidence of dopaminergic deficit. This latter group of patients is referred to as SWEDDs (Scans without Evidence of Dopamine Deficit) (Marek et al., 2011).

Some efforts based on deep learning have been done to design algorithms to help PD diagnosis. The Martinez-Murci team has continuously published a series of papers using deep learning techniques for PD diagnosis in a SPECT image dataset. Ortiz et al. (2016) designed a framework to automatically diagnose PD using deep sparse filtering-based features. Sparse filtering, based on ℓ_2_-norm regularization, extracted the suitable features that can be used as the weight of hidden layers in a three-layer DNN. Subsequently, this team firstly applied 3D-CNN in PD diagnosis. These methods achieved up to a 95.5% accuracy and 96.2% sensitively (Martinez-Murcia et al., 2017). However, this 3D-CNN architecture with only two convolutional layers was too shallow and limited the capability to extract more discriminative features. Martinez-Murcia et al. (2018) therefore proposed a deep convolutional AE (DCAE) architecture for feature extraction. The DCAE overcome two common problems: the need for spatial normalization and the effect of imbalanced datasets. For a strongly imbalanced (5.69/1) PD dataset, DCAE achieved more than 93% accuracy. Choi et al. (2017) developed a deep CNN model (PDNet) consisted of four 3D convolutional layers. PDNet obtained high classification accuracy compared to the quantitative results of expert assessment and can further classify the SWEDD and NC subjects. Esmaeilzadeh et al. (2018) both utilized the sMRI scans and demographic information (i.e., age and gender) of patients to train a 3D-CNN model. The proposed method firstly found that the *Superior* *Parietal* part on the right hemisphere of the brain was critical in PD diagnosis. Sivaranjini and Sujatha (2019) directly introduced the AlexNet model, which was trained by the transfer learned network. Shen et al. (2019b) proposed an improved DBN model with an overlapping group lasso sparse penalty to learn useful low-level feature representations. To incorporate multiple brain neuroimaging modalities, Zhang et al. (2018b) and McDaniel and Quinn (2019) both used a GCN model and presented an end-to-end pipeline without extra parameters involved for view pooling and pairwise matching. Transcranial sonography (TCS) had recently attracted increasing attention, and Shen et al. (2019a) proposed an improved DPN algorithm that embedded the empirical kernel mapping the network pruning strategy and dropout approach for the purposes of feature representation and classification for TCS-based PD diagnosis. Table 5 summarized each paper above reviewed for PD diagnosis.

**Table 5:** Overview of papers using deep learning techniques for PD diagnosis.

**References**	**Year**	**Database**	**Modality**	**Method**	**Modality**			**Accuracy (%)**	
					**PD**	**NC**	**SWEED**	**PD/NC**	**SWEED/NC**
Ortiz et al. (2016)	2016	PPMI	SPECT	DNN	–	–	–	95.0	–
Martinez-Murcia et al. (2017)	2017	PPMI	SPECT	3D-CNN	158	111	32	95.5 ± 4.4	82.0 ± 6.8
Choi et al. (2017)	2017	PPMI	SPECT	3D-CNN	431	193	77	96.0	76.5
		SNUH^a^	SPECT		72	10	–	98.8	–
Esmaeilzadeh et al. (2018)	2018	PPMI	sMRI + DI^e^	3D-CNN	452	204	–	1.0	–
Martinez-Murcia et al. (2018)	2018	PPMI	SPECT	DCAE	1,110	195	–	93.3 ± 1.6	–
Sivaranjini and Sujatha (2019)	2019	PPMI	SPECT	2D-CNN	100	82	–	88.9	–
Zhang et al. (2018b)	2018	PPMI	sMRI + DTI	GCNN	596	158	–	95.37 (AUC)	–
McDaniel and Quinn (2019)	2019	PPMI	sMRI + DTI	GCNN	117	30	–	92.14	–
Shen et al. (2019b)	2019	HSHU^b^	PET	DBN	100	200	–	90.0	–
		WXH^c^	PET		25	25	–	86.0	–
Shen et al. (2019a)	2019	Multi-site^d^	TCS	DPN	76	77	–	86.95 ± 3.15	–

a *SNUH, Seoul National University Hospital cohort*.

b *HSH, HuaShan Hospital cohort*.

c *WXH, WuXi 904 Hospital cohort*.

d *Shanghai East Hospital of Tongji University and the Second Affiliated Hospital of Soochow University*.

e *DI, Demographic Information*.

Up to now, only some papers have applied deep learning for PD diagnosis based on neuroimaging, and most of them adopt the 3D-CNN model. The traditional machine learning was still a popular and important technology for PD diagnosis, such as sparse feature learning (Lei et al., 2018), unsupervised learning (Singh and Samavedham, 2015), semi-unsupervised learning (Adeli et al., 2018), multi-task learning (Emrani et al., 2017), and classifier design (Shi et al., 2018).

### 3.3. Deep Learning for Austism Spectrum Disorder Analysis

Autism spectrum disorder (ASD) is a common neurodevelopmental disorder, which has affected 62.2 million ASD cases in the world in 2015. The Autism Imaging Data Exchange (ABIDE) initiative had aggregated rs-fMRI brain scans, anatomical and phenotypic datasets, collected from laboratories around the world. The ABIDE initiative included two large scale collections: ABIDE I and ABIDE II, which were released in 2012 and 2016, respectively. The ABIDE I collection involved 17 international sites and consisted of 1,112 subjects comprised of 539 from autism patients and 573 from NC. To further enlarge the number of samples with better-characterized, the ABIDE II collection involved 19 international sites, and aggregated 1,114 subjects from 521 individuals with ASD and 593 NC subjects (Di et al., 2014).

Many methods have been proposed on the application of deep learning for ASD diagnosis. These methods can be divided into three categories: AE-based methods, convolutional-based methods, and RNN-based methods. AE-based methods used various AE variations or stacked multiple AE to reduce data dimension and discovery highly discriminative representations. Hazlett et al. implemented the basic SAE, which primarily used surface area information from brain MRI at 6- and 12-months-old infants to predict the 24-months diagnosis of autism in children at high familial risk for autism. The SAE contained three hidden layers to reduce 315 dimension measurements to only two features (Hazlett et al., 2017). Two papers both used a stacked multiple sparse AE (SSAE) to learn low dimensional high-quality representations of functional connectivity patterns (Guo et al., 2017; Kong et al., 2019). But the difference was that Guo et al. input the whole-brain functional connectivity patterns and Kong et al. only selected the top 3,000 ranked connectivity features by F-score in descending order. Dekhil et al. (2018) built an automated autism diagnosis system, which used 34 sparse AE for 34 spatial activation areas. Each sparse AE extracted the power spectral densities (PSDs) of time courses in a higher-level representation and simultaneously reduced the feature vectors dimensionality. Choi (2017) used VAE to summarize the functional connectivity networks into two-dimensional features. One feature was identified using a high discrimination between ASD and NC, and it was closely associated with ASD-related brain regions. Heinsfeld et al. (2018) used DAE to reduce the effect of multi-site heterogeneous data and improve the generalization. Due to insufficient training samples, Li et al. (2018a) developed a novel deep neural network framework with the transfer learning technique for enhancing ASD classification. This framework was firstly trained an SSAE to learn functional connectivity patterns from healthy subjects in the existing databases. The trained SSAE was then transferred to a new classification with limited target subjects. Saeed et al. designed a data augmentation strategy to produce synthetic datasets needed for training the ASD-DiagNet model. This model was composed of an AE and a single-layer perceptron to improve the quality of extracted features (Saeed et al., 2019).

Due to collapsed the rs-fMRI scans into a feature vector, the above methods discarded the spatial structure of the brain networks. To fully utilize the whole brain spatial fMRI information, Li et al. (2018b) implemented 3D-CNN to capture spatial structure information and used sliding windows over time to measure temporal statistics. This model was able to learn ASD-related biological markers from the output of the middle convolution layer. Khosla et al. proposed a 3D-CNN framework for connectome-based classification. The functional connectivity of each voxel to various target ROIs was used as input features, which reserved the spatial relationship between voxels. Then the ensemble learning strategy was employed to average the different ROI definitions to reduce the effect of empirical selections, it and obtained more robust and accurate results (Khosla et al., 2018). Ktena et al. (2018) implemented a Siamese GCN to learn a graph-similarity metric, which took the graph structure into consideration for the similarity between a pair of graphs. This was the first application of metric learning with graph convolutions on brain connectivity networks. Parisot et al. (2017) introduced a spectral GCN for brain analysis in populations combining imaging and non-imaging information. The populations were represented as a sparse graph where each vertex corresponded to an imaging feature vector of a subject, and the edge weights were associated with phenotypic data, such as age, gender, and acquisition sites. Like the graph-based label propagation, a GCN model was used to infer the classes of unlabeled nodes on the partially labeled graphs. There existed no definitive method to construct reliable graphs in practice. Thus, Anirudh and Thiagarajan (2017) proposed a bootstrapped version of GCN to reduce the sensitivity of models on the initial graph construction step. The bootstrapped GCN used an ensemble of the weekly GCN, each of which was trained by a random graph. In addition, Yao et al. (2019) proposed a multi-scale triplet GCN to avoid the spatial limitation of a single template. A multi-scale templates for coarse-to-fine ROI parcellation were applied to construct multi-scale functional connectivity patterns for each subject. Then a triple GCN model was developed to learn multi-scale graph features of brain networks.

Several RNN-based methods were proposed to fully utilize the temporal information in the rs-fMRI time-series data. Bi et al. (2018) designed a random NN cluster, which combined multiple NNs into a model, to improve the classification performance in the diagnosis of ASD. Compared to five different NNs, the random Elman cluster obtained the highest accuracy. It is because that the Elman NN fit handling the dynamic data. Dvornek et al. (2017) first applied LSTM to ASD classification, which directly used the rs-fMRI time-series data, rather than the pre-calculated measures of brain functional connectively. The authors thought that the rs-fMRI time-series data contained more useful information of dynamic brain activity than single and static functional connectivity measures. For clarity, the important information of the above-mentioned papers was summarized in Table 6.

**Table 6:** Overview of papers using deep learning techniques for ASD diagnosis.

**References**	**Year**	**Database**	**Subject**		**Modality**	**Model**	**Accuracy (%)**
			**ASD**	**NC**			
Guo et al. (2017)	2017	ABIDE I	55	55	rs-fMRI	SSAE	86.36
Kong et al. (2019)	2019	ABIDE I	78	104	rs-fMRI	SSAE	90.39
Li et al. (2018a)	2018	ABIDE: UM^a^	48	65	rs-fMRI	SSAE	67.2
		ABIDE:UCLA^b^	36	39			62.3
		ABIDE: USM^c^	38	23			70.4
		ABIDE: LEUVEN^d^	27	34			68.3
Choi (2017)	2017	ABIDE	465	507	rs-fMRI	VAE	0.60 (AUC)
Heinsfeld et al. (2018)	2018	ABIDE	505	530	rs-fMRI	DAE	70.0
Hazlett et al. (2017)	2017	NDAR^e^	106	42	rs-fMRI	SAE	88.0
Dekhil et al. (2018)	2018	NDAR	123	160	rs-fMRI	SSAE	91.0 ± 3.2
Saeed et al. (2019)	2019	ABIDE	505	530	rs-fMRI	AE	70.1 ± 3.2
Li et al. (2018b)	2018	–	82	48	rs-fMRI	3D-CNN	89.0 ± 5.0 (F-score)
Khosla et al. (2018)	2018	ABIDE	542	625	rs-fMRI	3D-CNN	73.3
(Parisot et al., 2017)	2017	ABIDE	403	468	rs-fMRI	GCN	69.5
Anirudh and Thiagarajan (2017)	2017	ABIDE	404	468	rs-fMRI	GCN	70.8
Yao et al. (2019)	2019	ABIDE	438	544	rs-fMRI	GCN	67.3
Ktena et al. (2018)	2018	ABIDE	403	468	rs-fMRI	GCN	62.9
Dvornek et al. (2017)	2017	ABIDE	1,100	–	rs-fMRI	LSTM	68.5 ± 5.5
Bi et al. (2018)	2018	ABIDE	50	42	rs-fMRI	RNN	84.7 ± 3.2

a *University of Michigan*.

b *University of California, Los Angeles*.

c *University of Utah School of Medicine*.

d *Katholieke Universiteit Leuven*.

e *National Database of Autism Research*.

### 3.4. Deep Learning for Schizophrenia Analysis

Schizophrenia (SZ) is a prevalent psychiatric disorder and affects 1% of the population worldwide. Due to the complex clinical symptoms, the pathological mechanism of schizophrenia remains unclear and there is no definitive standard in the diagnosis of SZ. Different from the ADNI for AD diagnosis, the PPMI for PD diagnosis, and the ABIDE for ASD diagnosis, there was not a widely used neuroimaging dataset for the SZ diagnosis. Therefore, some studies have successfully applied source datasets that were available from the medical research centers, universities, and hospitals.

Recently, some studies have successfully applied deep learning algorithms to SZ diagnosis and have seen significant improvement. These methods were divided into two categories: unimodality and multi-modality, according to the types of input data, rather than according to deep learning architectures like AD or ASD diagnosis.

The unimodality category only used a single type of MRI and can furthermore be classified into subclasses: sMRI-methods and fMRI-methods. sMRI-methods discovery latent features from sMRI dataset, which can provide information on the tissue structure of the brain, such as gray matter, white matter, and cerebrospinal fluid. Plis et al. and Pinaya et al. used the DBN model, which only contained three hidden layers, to automatically extract feature for SZ identification. The results achieved a modestly higher predictive performance than the shallow-architecture SVM approach (Plis et al., 2014; Pinaya et al., 2016). Different from the DBN model in Pinaya et al. (2016), Pinaya et al. (2019) trained an SAE to create a normative model from 1,113 NC subjects, then used this model to estimate total and regional neuroanatomical deviation in individual patients with SZ. Ulloa et al. proposed a novel classification architecture that used synthetic sMRI scans to mitigate the effects of a limited sample size. To generate synthetic samples, a data-driven simulator was designed that can capture statistical properties from observed data using independent component analysis (ICA) and a random variable sampling method. Then a 10-layer DNN was trained exclusively on continuously generated synthetic data, and it greatly improves generalization in the classification of SZ patients and NC (Ulloa et al., 2015).

The fMRI-methods extracted discriminative features from rs-fMRI brain images with functional connectivity networks. Kim et al. (2015) learned lower-to-higher features via the DNN model in which each hidden layer was added *L*_1_-regularization to control the weight sparsity, and they also achieved 85.8% accuracy. Patel et al. used an SAE model with four hidden layers to separately train on each brain region. The input layer directly uses the complete time series of all active voxels without converting them into region-wise mean time series. This therefore ensured that the model retained more information (Patel et al., 2016). Due to the limited size of SZ dataset, Zeng et al. collected a large multi-site rs-fMRI dataset from seven neuroimaging resources. An SAE with an optimized discriminant item was designed to learn imaging site-shared functional connectivity features. This model can achieve accurate SZ classification performance across multiple independent imaging sites, and the learned features found that dysfunctional integration of the cortical-striatal-cerebellar circuit may play an important role in SZ (Zeng et al., 2018). Qureshi et al. built a 3D-CNN-based deep learning classification framework, which used the 3D ICA functional network maps as input. These ICA maps served as highly discriminative 3D imaging features for the discrimination of SZ (Qureshi et al., 2019). To exploit both spatial and temporal information, Dakka et al. and Yan et al. proposed a recurrent convolutional neural network involving CNN followed by LSTM and GRU, respectively. The CNN extracted spatial features, which then were fed to the followed RNN model to learn the temporal dependencies (Dakka et al., 2017; Yan et al., 2019).

Combined multi-modality brain images can improve the performance of disorder diagnosis. The MLSP2014 (Machine Learning for Signal Processing) SZ classification challenge provided 75 NC and 69 SZ, which both contained sMRI and rs-fMRI brain images. Qi and Tejedor (2016) used deep canonical correlation analysis (DCCA) and deep canonically correlated auto-encoders (DCCAE) to fuse multi-modality features. But in the proposed method, two modalities features directly were combined as 411 dimensional vector, then fed to the three-layer DNN model (Srinivasagopalan et al., 2019). To alleviate the missing modality, the synthetic sMRI and rs-fMRI images were generated by a generator proposed, and they were then used to train a multi-modality DNN (Ulloa et al., 2018). For clarity, the important information of the above-mentioned papers was summarized in Table 7. From this table, it can be seen the datasets for SZ diagnosis come from different universities, hospitals, and medical centers.

**Table 7:** Overview of papers using deep learning techniques for SZ diagnosis.

**References**	**Year**	**Database**	**Subject**		**Modality**	**Model**	**Accuracy (%)**
			**SZ**	**NC**			
Plis et al. (2014)	2014	Multi-site1^a^	198	191	sMRI	DBN	91.0 + 14 (F-score)
Ulloa et al. (2015)	2015	Multi-site1	198	191	sMRI	DNN	75.0 ± 4 (AUC)
Pinaya et al. (2016)	2016	UNIFESP^b^	143	83	sMRI	DBN	73.55 ± 6.84
Pinaya et al. (2019)	2019	NUSDAST^c^	30	40	sMRI	SAE	70.7
Kim et al. (2015)	2015	NITRC^d^	50	50	rs-fMRI	DNN	85.8
Patel et al. (2016)	2016	COBRE^e^	72	74	rs-fMRI	SAE	92.0
Zeng et al. (2018)	2018	Multi-site2^f^	357	377	rs-fMRI	SAE	85.0 ± 1.2
Qureshi et al. (2019)	2019	COBRE	72	74	rs-fMRI	3D-CNN	98.09 ± 1.01
Dakka et al. (2017)	2017	FBIRN^g^	46	49	rs-fMRI	CNN + LSTM	66.4
Yan et al. (2019)	2019	Multi-site3^h^	558	542	rs-fMRI	CNN + GRU	83.2 ± 3.2
Qi and Tejedor (2016)	2016	MLSP2014	69	75	sMRI + fMRI	DCCA/DCCAE	94.2/95.0 (AUC)
Srinivasagopalan et al. (2019)	2019	MLSP2014	69	75	sMRI + fMRI	DNN	94.44
Ulloa et al. (2018)	2018	FBIRN	135	169	sMRI + fMRI	DNN	85.0 ± 5.0 (AUC)

a *Johns Hopkins University; the Maryland Psychiatric Research Center; the Institute of Psychiatry; the Western Psychiatric Institute and Clinic at the University of Pittsburgh*.

b *the Universidade Federal de São Paulo*.

c *Northwestern University Schizophrenia Data and Software Tool*.

d *Neuroimaging Informatics Tools and Resources Clearinghouse website*.

e *Center for Biomedical Research Excellence*.

f *Xijing Hospital; First Affliated Hospital of Anhui Medical University; Second Xiangya Hospital; COBRE; the University of California, Los Angles and Washington University School of Medicine*.

g *The Function Biomedical Informatics Research Network Data*.

h *Peking University Sixth Hospital; Beijing Huilongguan Hospital; Xinxiang Hospital; Xinxiang Hospital; Xijing Hospital; Renmin Hospital of Wuhan University; Zhumadian Psychiatric Hospital*.

## 4. Discussion and Future Direction

As can be seen from this survey, consideration research has been reviewed on the subject of deep learning across four brain disorder diseases. Furthermore, the number of publications on medical imaging analysis shows an almost exponential growth in PubMed. Unfortunately, there is no unified deep learning framework that could be generally used for every disease research, even only for human disorder diseases. This is consistent with the “*No* *Free* *Lunch*” theorem, which states that there is no one model that works best for every problem. Thus, different deep learning methods are developed using different imaging modalities for a disease-specific task.

Although deep learning models have achieved great success in the field of neuroimaging-based brain disorder analysis, there are still some challenges that deserve further investigation. We summarize these potential challenges as follows and explore possible solutions.

First, deep learning algorithms highly depend on the configuration of hyper-parameter, which may dramatically fluctuate the performance. The hyper-parameter set composed of two parts: model optimization parameters (e.g., the optimization method, learning rate, and batch sizes, etc.) and network structure parameters (e.g., number of hidden layers and units, dropout rate, activation function, etc.). To obtain the best configuration, hyper-parameter optimization methods, including manual (e.g., grid search and random search) and automatic (e.g., Bayesian Optimization), are proposed. However, the method behind designing the architecture of deep neural networks still depends on the experienced experts. Recently, neural architecture search (NAS) automates this design of network architecture and indeed received new state-of-the-art performance (Zoph and Le, 2016; He et al., 2019). Additionally, another interesting technique called Population-Based Training (PTB), which is inspired by genetic algorithms, bridges and extends parallel search methods and sequential optimization methods. PBT is ability to automatic discovery of hyper-parameter schedules and model selection, which leads to stable training and better final performance (Jaderberg et al., 2017). It indicates that the hyper-parameter optimization may further mine the potential of deep learning in medical analysis.

Second, deep neural networks rely on complicated architectures to learn feature representations of the training data, and then makes its predictions for various tasks. These methods can achieve extremely accurate performances and may even beat human experts. But it is difficult to trust these predictions based on features you cannot understand. Thus, the black-box natural of the deep learning algorithms has restricted the practical clinical use. Some studies begin to explore the interpretability of deep learning in medical image analysis, and aim to show the features that most influence the predictions (Singh et al., 2020). An attention-based deep learning method is proposed and deemed as an interpretable tool for medical image analysis, which inspired by the way human pay attention to different parts of an image or the disease's influence on different regions of neuroimages (Sun et al., 2019b; Huang et al., 2020). The clinical diagnosis information as a modality is fused into the model to improve accuracy as well as give more comprehensive interpretability of outcomes (Hao et al., 2016, 2017; Wang et al., 2019a). Thus, how to improve the interpretability of deep learning model is worth further study and attention.

Third, deep learning methods require a large number of samples to train neural networks, though it is usually difficult to acquire training samples in many real-world scenarios, especially for neuroimaging data. The lack of sufficient training data in neuroimage analysis has been repeatedly mentioned as a challenge to apply deep learning algorithms. To address this challenge, a data augmentation strategy has been proposed, and it is widely used to enlarge the number of training samples (Hussain et al., 2017; Shorten and Khoshgoftaar, 2019). In addition, the use of transfer learning (Cheng et al., 2015, 2017) provides another solution by transferring well-trained networks on big sample datasets (related to the to-be-analyzed disease) to a small sample dataset for further training.

Fourth, the missing data problem is unavoidable in multimodal neuroimaging studies, because subjects may lack some modalities due to patient dropouts and poor data quality. Conventional methods typically discard data-missing subjects, which will significantly reduce the number of training subjects and degrade the diagnosis performance. Although many data-imputing methods have been proposed, most of them focus on imputing missing hand-crafted feature values that are defined by experts for representing neuroimages, while the hand-crafted features themselves could be not discriminative for disease diagnosis and prognosis. Several recent studies (Pan et al., 2018, 2019) propose that we directly impute missing neuroimages (e.g.,PET) based on another modality neuroimages (e.g.,MRI), while the correspondence between imaging data and non-imaging data has not been explored. We expect to see more deep network architectures in the near future to explore the association between different data modalities for imputing those missing data.

Fifth, an effective fusion of multimodal data has always been a challenge in the field. Multimodal data reflects the morphology, structure, and physiological functions of normal tissues and organs from different aspects and has strong complementary characteristics between different models. Previous studies for multimodal data fusion can be divided into two categories, *data-level fusion* (focus on how to combine data from different modalities) and *decision-level fusion* (focus on ensembling classifiers). Deep neural network architectures allow a third form of multimodal fusion, i.e., the intermediate fusion of learned representations, offering a truly flexible approach to multimodal fusion (Hao et al., 2020). As deep-learning architectures learn a hierarchical representation of underlying data across its hidden layers, learned representations between different modalities can be fused at various levels of abstraction. Further investigation is desired to study which layer of deep integration is optimal for problems at hand.

Furthermore, different imaging modalities usually reflect different temporal and spatial scales information of the brain. For example, sMRI data reflect minute-scale time scales information of the brain, while fMRI data can provide second-scale time scales information. In the practical diagnosis of brain disorder, it shows great significance for the implementation of early diagnosis and medical intervention by correctly introducing the spatial relationship of the diseased brain regions and other regions and the time relationship of the development of the disease progress (Jie et al., 2018; Zhang et al., 2018a). Although previous studies have begun to study the pathological mechanisms of brain diseases on a broad temporal and spatial scales, those methods usually consider either temporal or spatial characteristics (Wang et al., 2019b,d). It is therefore desirable to develop a series of deep learning frameworks to fuse temporal and spatial information for automated diagnosis of brain disorder.

Finally, the utilization of multi-site data for disease analysis has recently attracted increased attention (Heinsfeld et al., 2018; Wang et al., 2018, 2019c) since a large number of subjects from multiple imaging sites are beneficial for investigating the pathological changes of disease-affected brains. Previous methods often suffer from inter-site heterogeneity caused by different scanning parameters and subject populations in different imaging sites by assuming that these multi-site data are drawn from the same data distribution. Constructing accurate and robust learning models using heterogeneous multi-site data is still a challenging task. To alleviate the inter-site data heterogeneity, it could be a promising way to simultaneously learn adaptive classifiers and transferable features across multiple sites.

## 5. Conclusion

In this paper, we reviewed the most recent studies on the subject of applying the deep learning techniques in neuroimaging-based brain disorder analysis and focused on four typical disorders. AD and PD are both neurodegenerative disorders. ASD and SZ are neurodevelopmental and psychiatric disorders, respectively. Deep learning models have achieved state-of-the-art performance across the four brain disorders using brain images. Finally, we summarize these potential challenges and discuss possible research directions. With the clearer pathogenesis of human brain disorders, the further development of deep learning techniques, and the larger size of open-source datasets, a human-machine collaboration for medical diagnosis and treatment will ultimately become a symbiosis in the future.

## Author Contributions

DZ, ML, and LZ designed this review. LZ and MW searched the literatures. LZ wrote this manuscript. All authors read, edited, and discussed the article.

## Conflict of Interest

The authors declare that the research was conducted in the absence of any commercial or financial relationships that could be construed as a potential conflict of interest.
