# Supplementary material for: A Survey on Deep Learning for Neuroimaging-Based Brain Disorder Analysis
Source: Front Neurosci. 2020 Oct 8;14:779. doi: 10.3389/fnins.2020.00779 (PMC7578242; doi:10.3389/fnins.2020.00779)
Supplement: Supplementary file 1 [file Data_Sheet_1.pdf]

Supplementary Material

1 ABBREVIATIONS OF TERMINOLOGIES

For readers' convenience, the abbreviations of terminologies used in the following sections are listed in **Table S1**.

Table S1. Abbreviation of terminologies used in following sections.

Terminology	Abbr.	Terminology	Abbr.
Alzheimer's Disease	AD	Two-dimensional CNN	2D-CNN
Autism spectrum disorder	ASD	Three-dimensional CNN	3D-CNN
Computer-assisted diagnosis	CAD	Auto-encoder	AE
Converted MCI	cMCI	Artificial neural networks	ANN
Cerebrospinal fluid	CSF	Back-propagation	BP
Computed tomography	CT	Convolutional neural networks	CNN
Diffusion tensor imaging	DTI	Denoising auto-encoders	DAE
Electroencephalogram	EEG	Deep belief networks	DBN
Functional MRI	fMRI	Deep bolztman machine	DBM
Gray matter	GM	Deep generative model	DGM
Mild cognitive impairment	MCI	Deep neural networks	DNN
Magnetic resonance imaging	MRI	Deep polynomial network	DPN
Normal control	NC	Feed-forward neural network	FFNN
Parkinson's Disease	PD	Generative adversarial networks	GAN
Positron emission tomography	PET	Graph CNN	GCN
Region of interest	ROI	Gated recurrent unit	GRU
Resting-state fMRI	rs-fMRI	Long-short term memory	LSTM
Stable MCI	sMCI	Multi-layer perceptron	MLP
Structural MRI	sMRI	Principle component analysis	PCA
Single photon emission CT	SPECT	Restricted Bolztman machine	RBM
Schizophrenia	SZ	Recurrent neural networks	RNN
Transcranial Sonography	TCS	Region with CNN	R-CNN
White matter	WM	Stacked auto-encoders	SAE
		Stacked sparse AE	SSAE
		Support vector machine	SVM
		Variational auto-encoders	VAE

The abbreviations in the left column relate to the medical image analysis, and the abbreviations in the right column relate to the machine learning and deep learning architectures.

2 OPEN SOURCE DEEP LEARNING LIBRARY

With the great successes of deep learning techniques in various applications, some famous research groups and companies have released their source codes and tools in deep learning. Due to these open source toolkits, people are able to easily build deep models for their applications even if they are not acquainted with deep learning technique. **Table S2** lists the most popular toolkits for deep learning and shows their main features. All the software in the table can support for using GUP acceleration. For now, there are numerous deep learning toolkits available, but the problem it brings to people is how to select the most

Table S2. The popular open source toolkits for deep learning

Name	Creator	GitHub	License	Platform	Language	Interface
Caffe (Jia et al., 2014)	Berkeley Center	BVLC/caffe	BSD	Linux, macOS, Windows	C++	Python, MATLAB, C++
Deeplearning4j (Team et al., 2016)	Skymind	deeplearning4j/ deeplearning4j	Apache 2.0	Linux, macOS, Windows, Android	C++, Java	Java, Scala, Clojure, Python, Kotlin
Keras (Chollet and Others, 2015)	Franois Systems	fchollet/keras	MIT license	Linux, macOS, Windows	Python	Python, R
MXNet (Chen et al., 2015)	Apache Software Foundation	apache/ incubator-mxnet	Apache 2.0	Linux, macOS, Windows, AWS, Android, iOS, JavaScript	C++	C++, Python, Julia, Matlab, JavaScript, Go, R, Scala, Perl
TensorFlow (Abadi et al., 2016)	Google	tensorflow/ tensorflow	Apache 2.0	Linux, macOS, Windows, Android	C++, Python	Python, C, C++, Java, Go, JavaScript, R, Julia, Swift
PyTorch (Paszke et al., 2017)	Adam Paszke et al	pytorch/ pytorch	BSD	Linux, macOS, Windows	Python, C	Python
Theano (Alrfou et al., 2016)	Université de Montréal	Theano/ Theano	BSD	Linux, macOS, Windows	Python	Python
Torch (Ronan et al., 2011)	Ronan Collobert et al	torch/ torch7	BSD	Linux, macOS, Windows, Android, iOS	C, Lua, LuaJIT	C,Lua, LuaJIT
CNTK (Seide and Agarwal, 2016)	Microsoft	Microsoft/ CNTK	MIT license	Linux, macOS, Windows	C++	Python, C++, C#, Java
MATLAB	MathWorks	-	Proprietary	Linux, macOS, Windows	C,C++, Java, MATLAB	MATLAB

suitable toolkit for their applications. Selecting the best toolkit depends on the goals of the projects, the characters of the available dataset, the skills and background of the researchers, the features of the available toolkits Erickson et al. (2017); Zacharias et al. (2018). Therefore, when a project starts, it is worth spending time to evaluate candidate toolkits to be sure that the best suitable toolkit is chosen for the corresponding application.

3 TAXONOMY OF DEEP LEARNING METHODS

In this survey, we defined a taxonomy for the reviewed papers as shown in Figure S1. For the specific disease, we usually divided the proposed models into two or three categories. If one category has few papers, we will merge it and its sibling categories into their parent category. For AD diagnosis in this survey, if we divided these models into three categories: RBM-based, AE-based and CNN-based. But the RBM-based category only contained two papers. Thus, we merged the RBM-based and AE-based into the parent category: DGM-based.

REFERENCES

Abadi, M., Agarwal, A., Barham, P., Brevdo, E., Chen, Z., Citro, C., et al. (2016). TensorFlow: Large-Scale Machine Learning on Heterogeneous Distributed Systems

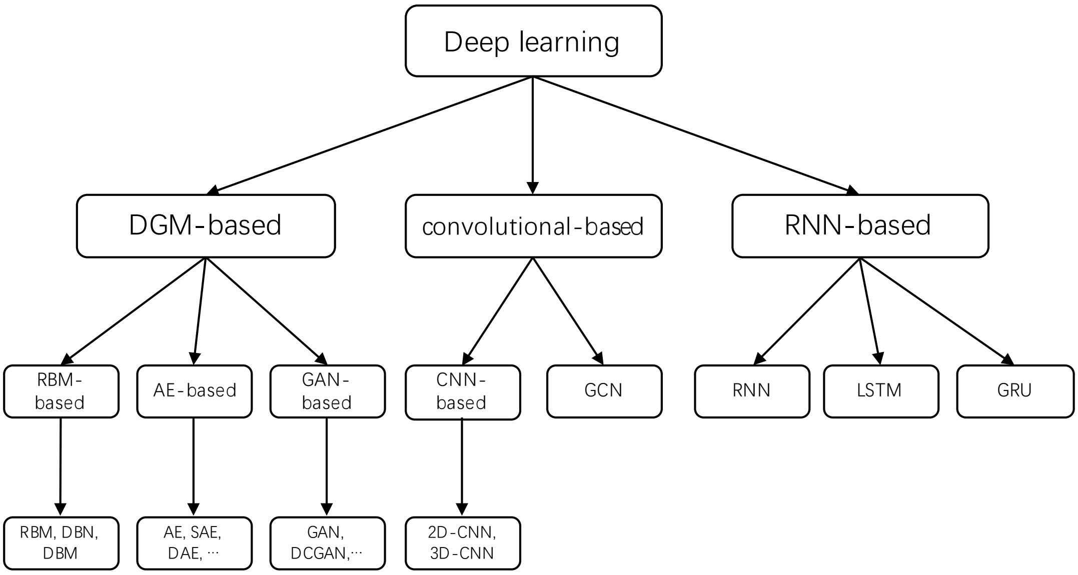

Figure S1. Taxonomy of deep learning methods in this survey.

Alrfou, R., Alain, G., Almahairi, A., Angermueller, C., Bahdanau, D., Ballas, N., et al. (2016). Theano: A Python framework for fast computation of mathematical expressions. *arXiv: Symbolic Computation*

Chen, T., Li, M., Li, Y., Lin, M., Wang, N., Wang, M., et al. (2015). MXNet: A Flexible and Efficient Machine Learning Library for Heterogeneous Distributed Systems. *CoRR* abs/1512.01274

Chollet, F. and Others (2015). *Keras*. https://keras.io

Erickson, B. J., Korfiatis, P., Akkus, Z., Kline, T., and Philbrick, K. (2017). Toolkits and libraries for deep learning. *Journal of digital imaging* 30, 400–405

Jia, Y., Shelhamer, E., Donahue, J., Karayev, S., Long, J., Girshick, R., et al. (2014). Caffe: Convolutional architecture for fast feature embedding. In *Acm International Conference on Multimedia*

Paszke, A., Gross, S., Chintala, S., Chanan, G., Yang, E., DeVito, Z., et al. (2017). Automatic differentiation in PyTorch

Ronan, C., Kavukcuoglu, K., and Farabet, C. (2011). Torch7: A matlab-like environment for machine learning. In *BigLearn, NIPS workshop*

Seide, F. and Agarwal, A. (2016). CNTK: Microsoft's Open-Source Deep-Learning Toolkit. In *Proceedings of the 22Nd ACM SIGKDD International Conference on Knowledge Discovery and Data Mining* (New York, NY, USA: ACM), 2135–2135. doi:10.1145/2939672.2945397

Team, D. et al. (2016). Deeplearning4j: Open-source distributed deep learning for the jvm. *Apache Software Foundation License* 2

Zacharias, J., Barz, M., and Sonntag, D. (2018). A Survey on Deep Learning Toolkits and Libraries for Intelligent User Interfaces. *arXiv preprint arXiv:1803.04818*
